# Presence of multiple genotypes in subjects with HPV-16 infection is highly associated with anal squamous intraepithelial lesions in HIV-1 infected males

**DOI:** 10.1371/journal.pone.0186367

**Published:** 2017-10-31

**Authors:** Cristina Rovelli, Andrea Poli, Laura Galli, Massimo Cernuschi, Andrea Marco Tamburini, Sara Racca, Giuseppe Tambussi, Serena Rolla, Luca Albarello, Riccardo Rosati, Adriano Lazzarin, Antonella Castagna, Silvia Nozza

**Affiliations:** 1 Università Vita-Salute San Raffaele, Milan, Italy; 2 Infectious Diseases Department, San Raffaele Scientific Institute, Milan, Italy; 3 Gastrointestinal Surgery, San Raffaele Scientific Institute, Milan, Italy; 4 Laboratory of Microbiology, San Raffaele Scientific Institute, Milan, Italy; 5 Department of Pathology, San Raffaele Scientific Institute, Milan, Italy; Georgetown University, UNITED STATES

## Abstract

**Objectives:**

The aim of the study was to determine the prevalence of abnormal cytological findings, high risk (HR)-HPV genotypes and to identify factors associated with an abnormal cytological findings in a cohort of HIV-infected males.

**Patients and methods:**

Retrospective observational study on HIV-infected male patients who performed screening in the absence of clinical symptoms. Cytological abnormalities were classified as atypical squamous cells of undetermined significance (ASC-US), low-grade(LSIL) or high high-grade squamous intraepithelial lesion (HSIL). Logistic regression models were used to identify predictors of having LSIL/HSIL.

**Results:**

Among 875 pts, abnormal cytology findings were observed in 254 (29%, 95% CI: 26.1%-32.1%) subjects: 142 (16%) had LSIL and 49 (6%) HSIL. Overall, 581 (66%, 95%CI: 63.2%-69.5%) subjects had ≥1 HR-HPV type and 269 (31%) had ≥2 HR HPV types. Multivariate logistic regression showed that subjects with multiple HR-HPV genotypes (OR = 1.351, 95%CI: 1.005–2.111) and with HPV-16 type (OR = 2.032, 95%CI: 1.313–3.146) were more likely to have LSIL/HSIL in addition to a lower CD4+/CD8+ ratio, a previous diagnosis of syphilis and a positive viral load. In another multivariate model, the presence of multiple HPV types in subjects with HPV-16 type was associated with the highest adjusted OR of having a LSIL/HSIL (OR = 2.598, 95%CI: 1.460–4.624).

**Conclusions:**

In HIV-infected men, the prevalence of abnormal cytological findings was of 29% and of HR-HPV was 66%. The concomitant presence of HPV-16 and multiple HR genotypes was associated with an increased risk of abnormal cytological findings.

These data highlight the importance of screening multiple HPV genotypes in HIV-infected patients.

## Introduction

HPV is a necessary cause of the cervical cancer and it has an important role in the development of other cancers, especially of the head and neck and of the anogenital area [1]. HIV-infected patients have an increased risk for HPV infection and related lesions, due to lower specific immunological response and to viral interactions [2–4].

Anal High Grade Squamous Intraepithelial Lesions (HSIL) and anal cancer incidence have continued to increase in HIV infected men and they are specifically related to HPV-16 [5].

In a previous study on an Italian cohort of HIV-infected males who have sex with male (MSM), the authors found that 163 (84.5%) subjects had a multiple HPV genotypes infection and that HPV-16 was the most frequent genotype [6, 7].

The aim of this study was to determine the prevalence of abnormal cytological findings, high risk HPV (HR-HPV) genotypes and to identify the factors associated with an abnormal cytological finding in a large cohort of HIV-infected males.

## Methods

This is a retrospective observational study on HIV-infected male patients followed at the Department of Infectious Diseases of San Raffaele Scientific Institute who performed an HPV screening test for multiple HPV genotypes in the absence of clinical symptoms. Screening is performed using anal cytological exams with direct tissue evaluation and biopsy *via* high resolution anoscopy. This study was approved by the Ethics Committee of the San Raffaele Scientific Institute, and examined demographic, clinical and laboratory data obtained from the Infectious Diseases Department database of the San Raffaele Hospital (IDD-HSR). This observational database collects demographic, clinical, therapeutic, and laboratory data from adult patients who are receiving primary inpatient or outpatient care for HIV infection at the Infectious Diseases Department of the San Raffaele Scientific Institute (Milan, Italy). At their first visit to the clinic, patients provide written informed consent for scientific analysis of their clinical and laboratory data. Information regarding the prescribed antiretroviral and concomitant drugs (type, dosage, and dates of start and stop) are prospectively recorded at each visit by the treating physician, and these data are subsequently checked by skilled data managers. However, patient adherence to the prescribed drugs is not routinely assessed.

With regard to the HPV screening test, the following HPV genotypes were tested with Anyplex II HPV28 [8]. Based on Seegene’s proprietary DPO and TOCE technologies, this homogenous assay performs on real time PCR instruments to detect and differentiate 19 high-risk (16, 18, 26, 31, 33, 35, 39, 45, 51, 52, 53, 56, 58, 59, 66, 68, 69, 73, 82 genotypes) and 9 low-risk (6, 11, 40, 42, 43, 44, 54, 61, 70 genotypes) HPV[8]. Specimens for anal HPV determination were collected using a swab inserted 3–5 cm into the anus (approximate depth of the squamocolumnar junction), then rotated and placed into a transport tube with 2,5 mL Transport Buffer (guanidine thiocyanate in a Tris buffer). If more than one cytological test per patient was available (n = 87 patients), the analyses considered only the most recent finding.

Anal specimens for cytological (PAP smear) analysis were collected by study physicians, who introduced a swab into the anal canal and circularly rotated it. The swabs were then fixed on glass label with a liquid based cytology preparation. Anal cytology findings were classified as normal if the finding was reported as negative and abnormal if reported as atypical squamous cells of undetermined significance (ASC-US), low-grade(LSIL) or high high-grade squamous intraepithelial lesion (HSIL). Patients with LSIL and HSIL performed a surgical visit with high resolution anoscopy. The surgeon could have performed biopsies with histological examination based on the presence of lesions. Results of biopsies were defined according to Northfelt’s classification [9]: intraepithelial neoplasia in the anal canal (AIN) was divided into three grades: AIN1, AIN2 and AIN3; grade 1 is defined as nuclear abnormalities confined to the lower third of the epithelium, grade 2 to the lower two-thirds, and grade 3 through the full thickness of the epithelium.

Results were described with median (IQR) or frequency (%). Chi-square and Kruskal-Wallis or Mann-Whitney tests (as appropriate) were used to evaluate differences between groups in categorical and continuous variables, respectively. Prevalence rates were reported with the corresponding two-sided 95% confidence interval (CIs) calculated using the Wald method.

Linear trends in proportions were assessed by use of the Cochran-Armitage trend test.

Univariate and multivariate logistic regression models were used to identify factors associated with having an abnormal cytological finding (either LSIL/HSIL [Model 1,2,3] or ASC-US/LSIL/HSIL [Model 4, 5,6]) reported as odds ratios (OR) and 95% confidence intervals (CI). Age, HIV risk factor, years since first HIV positive test were chosen a priori as notably known to be predictive of an abnormal HPV cytological finding [2,3]; variables with a univariate p-value≤0.20 were also included into the multivariate models. To avoid multicollinearity among immunological covariates with an univariate p-value≤0.20 [CD4+, CD4% and CD4+/CD8+ ratio], only CD4+/CD8+ ratio was included in the multivariate models. Two further multivariate models, including a new covariate assessing the joint effect of HPV-16 type and multiple HPV types [i.e., HPV-16—and number of HPV types<2 (reference class), HPV-16—and number of HPV types≥2, HPV-16 + and number of HPV types<2, HPV-16 + and number of HPV types≥2] were also calculated on the risk of having an ASC-US/LSIL/HSIL or a LSIL/HSIL cytological finding.

A two-sided alpha level of 0.05 was taken as reference to detect statistical significance in the univariate and multivariate analyses. All analyses were conducted using SAS statistical software version 9.2 (Statistical Analyses System Inc, Cary, NC,USA).

## Results

A total of 875 pts were screened. Baseline patients characteristics were summarized in Table 1; 98% pts were treated with antiretroviral therapy (ART) since 5.2 (1.2–13.8)years, 82% had HIV-RNA<50 cps/mL.

**Table 1:** Patients’ characteristics.

	ALL (n = 875)	No lesion (n = 621)	ASC-US (n = 63)	LSIL (n = 142)	HSIL (n = 49)	P-value
Age *(years)*	43.1 (35.9–50.1)	43.2 (36.4–50.3)	42.2 (34.1–49.7)	42.3 (34.9–48.6)	43.4 (35.9–52.0)	0.279
HIV Risk factor						0.698
Men who have sex with men	696 (80%)	494 (80%)	49 (78%)	111 (78%)	42 (86%)	
Other	179 (20%)	127 (20%)	14 (22%)	31 (22%)	7 (14%)	
Years since first HIV positive test	7.9 (2.5–16.3)	8.3 (2.7–16.6)	4.4 (1.1–14.7)	6.7 (2.4–15.1)	9.9 (4.7–17.8)	0.025
Naïve	15 (1.7%)	10 (1.6%)	2 (3.1%)	3 (2.1%)	0	0.609
Duration of HAART (*years*)	5.2 (1.2–13.8)	5.8 (1.7–14.1)	2.1 (0.7–9.0)	3.7 (0.9–13.1)	6.7 (1.0–15.9)	0.004
Previous syphilis infection	490 (56%)	329 (53%)	37 (59%)	87 (61%)	37 (76%)	0.009
Ab anti-HCV						0.839
Negative	739 (84%)	523 (84%)	56 (89%)	119 (84%)	41 (84%)	
Positive	113 (13%)	80 (13%)	6 (10%)	21 (15%)	6 (12%)	
Unknown	23 (3%)	18 (3%)	1 (1%)	2 (1%)	2 (4%)	
HBsAg						0.325
Negative	668 (76%)	476 (77%)	48 (76%)	110 (78%)	34 (69%)	
Positive	55 (6%)	45 (7%)	2 (3%)	6 (4%)	2 (4%)	
Unknown	152 (17%)	100 (16%)	13 (21%)	26 (18%)	13 (27%)	
Previous AIDS diagnosis	89 (10%)	57 (9%)	4 (6%)	14 (10%)	14 (29%)	0.0002
Nadir CD4+ *(cells/μL)*	319 (221–456)	319 (223–457)	367 (183–493)	308 (225–442)	308 (153–450)	0.832
CD4+ *(cells/μL)*	675 (522–874)	682 (539–880)	637 (450–835)	667 (492–831)	643 (455–838)	0.122
CD4%	29.6 (24.5–35.0)	30.2 (25.9–35.8)	27.7 (23.0–33.3)	28.1 (23.0–33.4)	26.0 (20.4–33.7)	<0.0001
CD4+/CD8+ ratio	0.73 (0.51–1.01)	0.76 (0.54–1.04)	0.63 (0.40–0.88)	0.63 (0.47–0.93)	0.57 (0.38–0.91)	0.0002
Pre-ART HIV-RNA *(log*_*10*_ *cps/mL)*	4.89 (4.21–5.32)	4.88 (4.13–5.34)	4.96 (4.49–5.37)	4.86 (4.30–5.26)	4.96 (4.20–5.45)	0.670
HIV-RNA <50 cps/mL	711(82%)	520 (85%)	44 (70%)	111 (78%)	36 (73%)	0.004
HPV-16 genotype	237 (27%)	143 (23%)	16 (25%)	52 (37%)	26 (53%)	<0.0001
HPV-18 genotype	118 (14%)	62 (10%)	15 (24%)	28 (20%)	13 (27%)	0.0004
HPV-6/11 genotypes	96 (11%)	47 (8%)	12 (19%)	28 (20%)	9 (18%)	<0.0001
HPV-31 genotype	27 (3%)	13 (2%)	4 (6%)	5 (4%)	5 (10%)	<0.0001
HPV-33 genotype	61 (7%)	41 (7%)	7 (11%)	9 (6%)	4 (8%)	0.543
HPV-35 genotype	63 (7%)	41 (7%)	7 (11%)	10 (7%)	5 (10%)	0.410
HPV-39 genotype	40 (5%)	26 (4%)	8 (13%)	6 (4%)	0	0.021
HPV-45 genotype	38 (4%)	23 (4%)	4 (6%)	6 (4%)	5 (10%)	0.166
HPV-51 genotype	42 (5%)	24 (4%)	7 (11%)	7 (5%)	4 (8%)	0.062
HPV-52 genotype	37 (4%)	25 (4%)	7 (11%)	5 (4%)	0	0.055
HPV-56 genotype	30 (3%)	19 (3%)	3 (5%)	7 (5%)	1 (2%)	0.562
HPV-58 genotype	107 (12%)	75 (12%)	6 (10%)	18 (13%)	8 (16%)	0.512
HPV-59 genotype	34 (4%)	18 (3%)	6 (10%)	7 (5%)	3 (6%)	0.058
HPV-68 genotype	73 (8%)	55 (9%)	7 (11%)	8 (6%)	3 (6%)	0.686
HPV-73 genotype	48 (5%)	28 (5%)	6 (10%)	10 (7%)	4 (8%)	0.214

A normal cytological result was found in 621(71%) subjects while abnormal cytology findings were observed in 254 (29%, 95% CI: 26.1%-32.1%) subjects. Sixty-three (7.2%, 95%CI: 5.7%-9.1%) showed atypical squamous cells of undetermined significance (ASC-US), 142 (16%, 95%CI: 13.9%-18.8%) had LSIL and 49 (5.6%, 95%CI: 4.3%-7.3%) had HSIL. A cytologic HSIL diagnosis was more frequent among pts with a previous diagnosis of AIDS [14 (29%) vs 75 (9%), p<0.0001]; similarly, HSIL was more frequent among pts who had a previous diagnosis of syphilis rather than those without [37 (76%) vs 453(55%), p = 0.017].

Overall, 581 (66%, 95%CI: 63.2%-69.5%) subjects had ≥1 HPV type and 269 (31%, 95%CI: 27.8%-33.9%) had ≥2 HPV types.

Subjects with multiple HR-HPV genotypes showed a lower CD4+/CD8+ ratio [0.57 (0.38–0.91) vs 0.73 (0.52–1.02), p = 0.008] and were more frequently on ART [268 (99.6%) vs 592 (97.7%), p = 0.047], less frequently co-infected with HBV [4 (1.5%) vs 51 (8.4%), p = 0.0001] and with a previous diagnosis of AIDS [19 (7%) vs 70 (12%), p = 0.049] than those with ≤1 HR-HPV genotypes.

**Fig 1:**
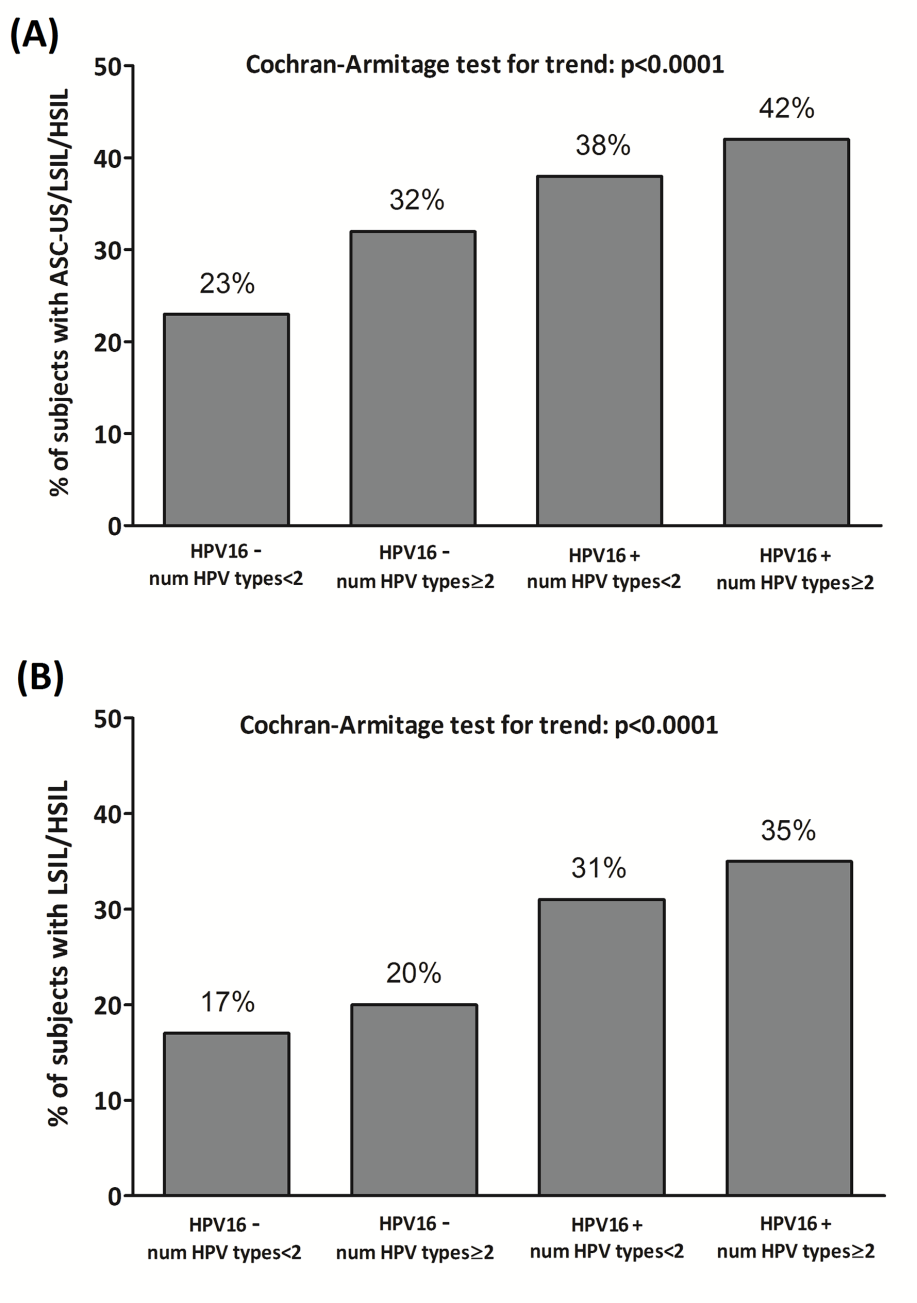
Joint effects of the occurrence of HPV-16 type and multiple HR-HPV genotypes on ASC-US, LSIL and HSIL (Panel A) and LSIL and HSIL (Panel B).

The proportion of subjects with an abnormal cytological finding (either LSIL/HSIL or ASC-US/LSIL/HSIL) were significantly different when considering the joint effects of the occurrence of HPV-16 type and multiple HR-HPV genotypes (p<0.0001 for both outcomes; Fig 1).

**Fig 2:**
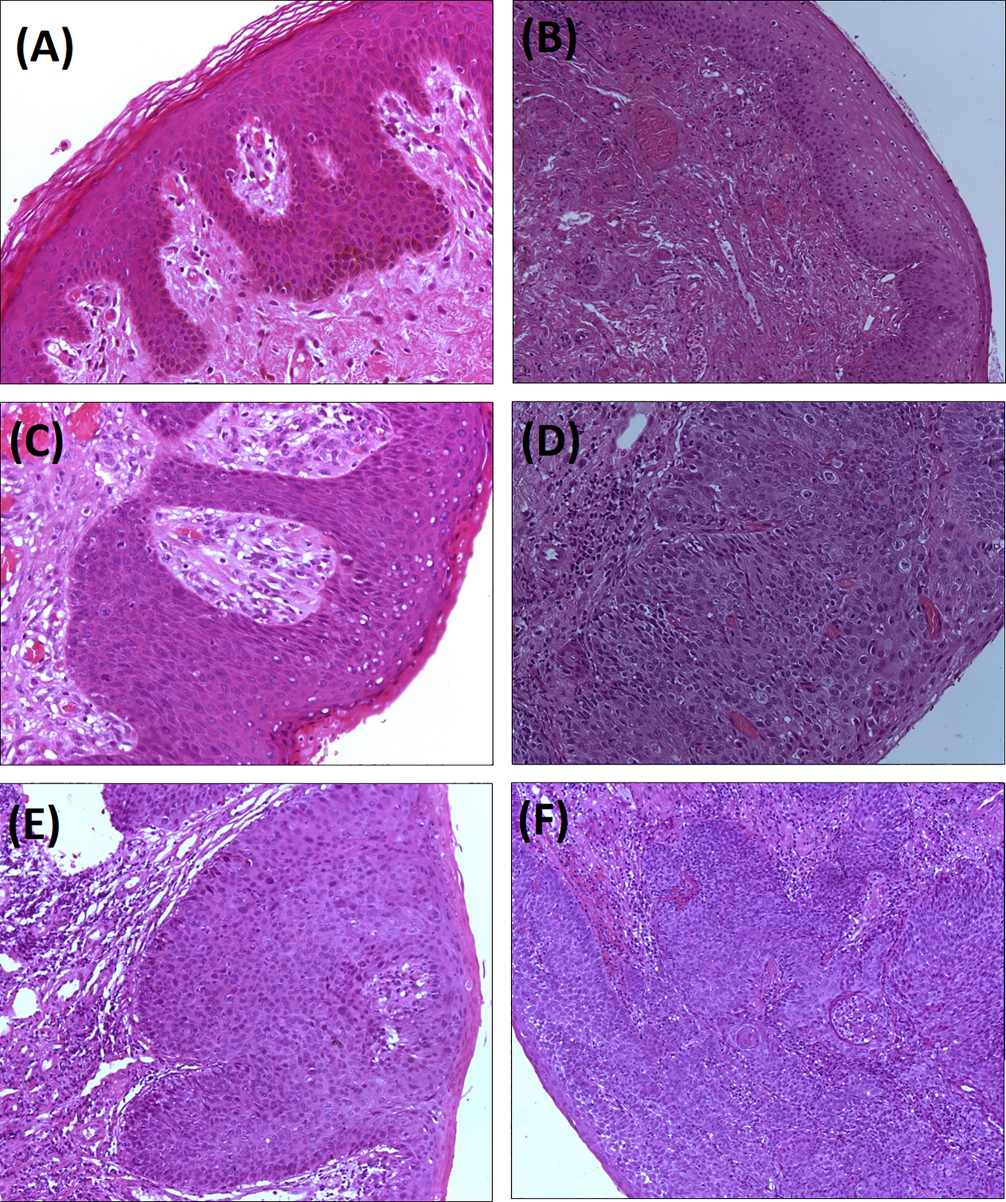
Histological images of patients with none, low- or high-grade intraepithelial neoplasia. (A) Negative for AIN lesion; anal skin with normal histology. (B) Negative for AIN lesion; anal skin with normal histology. (C) Anal Intraepithelial Neoplasia with low grade dysplasia (LSIL). (D) Anal Intraepithelial Neoplasia with high grade dysplasia (HSIL). (E) Anal Intraepithelial Neoplasia with high grade dysplasia (HSIL). (F) Anal Intraepithelial Neoplasia with high grade dysplasia (HSIL, left) with an extensive area of squamous cell carcinoma infiltrating the stroma (middle and right).

Eighty-two (9.4%) subjects also performed a histological test: 5 (6%) patients had no intraepithelial neoplasia, 4 (5%) had a low-grade (grade 1) squamous intraepithelial neoplasia of the anus, 62 (76%) %) had a high-grade squamous intraepithelial neoplasia [44 (54%) of grade 2 and 18 (22%) of grade 3] and 11 (13%) patients had a diagnosis of anal cancer. Histological images of patients with none, low- or high-grade intraepithelial neoplasia are shown in Fig 2.

The characteristics of the 11 patients with an anal cancer diagnosis are shown in Table 2; among them, 10 (91%) subjects showed a HSIL cytologic finding and the HPV-16 type was the most prevalent (n = 10, 91%).

**Table 2:** Characteristics of patients with a diagnosis of anal cancer.

ID	Cytologic finding	Age	NADIR CD4+	Previous diagnosis of AIDS	HCVAB	HBSAG	Previous diagnosis of syphylis	Years since HIV diagnosis	HAART duration (years)	HIV-RNA	CD4+/CD8+ ratio	CD4+	Number of HPV types	HPV types
1295	HSIL	58,9	233	0	neg	neg	neg	18	17.9	<50 cps/mL	0.57	683	3	18, 31, 35
2247	HSIL	52,8	277	0	neg	neg	pos	17.1	17	<50 cps/mL	0.80	416	1	16
2479	LSIL	56,0	191	0	pos	neg	neg	25.4	18.4	<50 cps/mL	0.54	939	2	16,11
2524	HSIL	65,8	657	1	neg	neg	pos	17.1	16.9	≥50 cps/mL	0.49	657	1	16
2552	HSIL	40,2	130	1	neg	neg	pos	17.7	16.5	<50 cps/mL	1.07	1312	1	16
4077	HSIL	51,9	45	1	neg	neg	neg	17.9	15.9	≥50 cps/mL	0.20	482	1	16
5286	HSIL	47,0	353	0	neg	ukn	neg	25.1	12.2	<50 cps/mL	0.57	391	1	16
5759	HSIL	57,6	372	0	neg	ukn	neg	21.1	17.8	<50 cps/mL	0.37	552	1	16
6044	HSIL	52,4	230	0	neg	neg	neg	22.2	21.7	<50 cps/mL	0.46	848	2	16, 11
8623	HSIL	54,5	34	1	neg	neg	neg	1.1	1	<50 cps/mL	0.22	148	4	16, 18, 56, 11
9817	HSIL	48,2	112	0	neg	neg	pos	25.5	24.9	<50 cps/mL	0.22	134	4	16, 31, 45, 73

Results of the univariate and multivariate logistic regression models on the risk of a LSIL/HSIL cytologic finding are summarized in Table 3 (Model 1): subjects with a lower CD4+/CD8+ ratio, with multiple HR-HPV genotypes and with a previous diagnosis of syphilis or AIDS were more likely to have LSIL/HSIL. Findings didn’t modify when the number of HPV genotypes was included as a continuous variable [adjusted OR_per 1 more genotype_: 1.31, 95% CI: 1.16 to 1.48, p<0.0001].

**Table 3:** Univariate and multivariate logistic regression analyses on the risk of having a LSIL/HSIL cytologic finding.

	Univariate analysis		Multivariate analysis: Model 1		Multivariate analysis: Model 2		Multivariate analysis: Model 3	
	Crude OR (95%CI)	p-value	Adjusted OR (95%CI)	p-value	Adjusted OR (95%CI)	p-value	Adjusted OR (95%CI)	p-value
Age (*per 5-years older)*	1.046 (0.967–1.133)	0.266	0.982 (0.862–1.116)	0.787	0.990 (0.868–1.127)	0.885	0.990 (0.868–1.127)	0.881
HIV risk factor							)	
other vs MSM	0.915 (0.550–1.470)	0.829	1.277 (0.651–2.506)	0.477	1.284 (0.649–2.540)	0.473	1.318 (0.684–2.615	0.430
Years of HIV infection (*per 5-years longer*)	1.022 (0.925–1.133)	0.668	0.953 (0.804–1.129)	0.581	0.956 (0.805–1.134)	0.607	0.957 (0.805–1.135)	0.613
ART status								
Naive vs treated	0.894 (0.202–2.848)	0.863	Not included		Not included		Not included	
Duration of HAART (*per 5-years longer*)	1.053 (0.939–1.184)	0.384	Not included		Not included		Not included	
Ab anti-HCV	)							
Positive vs Negative	1.136 (0.702–1.789	0.592	Not included		Not included		Not included	
HBsAg								
Positive vs Negative	0.619 (0.266–1.271)	0.193	0.718 (0.317–1.630)	0.429	0.745 (0.305–1.628)	0.485	0.753 (0.307–1.650)	0.503
Previous AIDS diagnosis								
Yes vs No	1.754 (1.073–2.809)	0.022	1.828 (0.966–3.458)	0.048	1.817 (0.952–3.469)	0.070	1.809 (0.946–3.460)	0.073
Previous syphilis infection								
Positive vs Negative	1.608 (1.156–2.252)	0.005	1.863 (1.192–2.910)	0.006	1.762 (1.122–2.766)	0.014	1.744 (1.110–2.739)	0.016
Nadir CD4+ *(per 50-cells/μL higher)*	0.995 (0.958–1.032)	0.783	Not included		Not included		Not included	
HIV-RNA zenith	0.985 (0.860–1.133)	0.826	Not included		Not included		Not included	
*(per log*_*10*_ *copies/mL higher)*								
CD4+ *(per 50-cells/μL higher)*	0.971 (0.942–0.999)	0.045	Not included		Not included		Not included	
CD4% *(per 10% higher)*	0.725 (0.600–0.875)	0.0008	Not included		Not included		Not included	
CD4+/CD8+ ratio (*per 0*.*1-point higher*)	0.916 (0.869–0.962)	0.0007	0.917 (0.861–0.972)	0.005	0.924 (0.866–0.979)	0.011	0.923 (0.865–0.979)	0.010
HIV-RNA								
≥50 vs <50 copies/mL	1.508 (1.010–2.222)	0.041	1.818 (1.066–3.096)	0.028	1.761 (1.025–3.021)	0.041	1.767 (1.029–3.040)	0.039
Number of HPV types (per 1-more type)	1.292 (1.175–1.422)	<0.0001						
≥2 vs <2	1.451 (1.034–2.028)	0.030	1.597 (1.037–2.457)	0.034	1.351 (1.005–2.111)	0.050	Not included	
HPV-16 type			Not included					
Yes vs No	2.262 (1.613–3.175)	<0.0001			2.032 (1.313–3.146)	0.002	Not included	
Number of HPV types and HPV-16 type		<0.0001	Not included		Not included			0.002
HPV-16 –and num HPV types<2	Ref	Ref					Ref	Ref
HPV-16 –and num HPV types≥2	1.223 (0.772–1.938)	0.093					1.553 (0.873–2.762)	0.164
HPV-16 + and num HPV types<2	2.196 (1.399–3.447)	0.042					2.337 (1.320–4.138)	0.046
HPV-16 + and num HPV types≥2	2.589 (1.648–4.065)	0.005					2.598 (1.460–4.624)	0.040

Abbreviation: ART, antiretroviral therapy; MSM, men who have sex with men; HAART, highly active antiretroviral therapy.

Age, HIV risk factor, years of HIV infection and other covariates with a p-value<0.20 at univariate analysis were included in the multivariate analysis.

In an additional multivariate model [Table 3, Model 2], subjects with the HPV-16 genotype were more likely to have a LSIL/HSIL cytological finding (p = 0.002) and those with multiple HPV types tended to be at higher risk of the same outcome (p = 0.087).

Finally, in a further multivariate model [Table 3] assessing the joint effect of HPV-16 type and multiple HPV types, the adjusted OR of having a LSIL/HSIL cytological finding progressively increased along categories (overall p = 0.002).

Similar results were found when evaluating the risk of an ASC-US/LSIL/HSIL cytologic finding (Table 4).

**Table 4:** Univariate and multivariate logistic regression analyses on the risk of having a ASC-US/LSIL/HSIL cytologic finding.

	Univariate analysis		Multivariate analysis: Model 4		Multivariate analysis: Model 5		Multivariate analysis: Model 6	
	Crude OR (95%CI)	p-value	Adjusted OR (95%CI)	p-value	Adjusted OR (95%CI)	p-value	Adjusted OR (95%CI)	p-value
Age (*per 5-years older)*	0.931 (0.865–1.001)	0.053	0.964 (0.854–1.088)	0.557	0.970 (0.858–1.096)	0.630	0.970 (0.857–1.095)	0.620
HIV risk factor								
other vs MSM	0.927 (0.595–1.442)	0.736	1.384 (0.737–2.599)	0.312	1.392 (0.737–2.629)	0.308	1.479 (0.780–2.803)	0.230
Years of HIV infection (*per 5-years longer*)	0.889 (0.798–0.988)	0.031	0.891 (0.757–1.046)	0.161	0.891 (0.756–1.048)	0.167	0.893 (0.758–1.051)	0.175
ART status								
Naive vs treated	1.227 (0.379–3.489)	0.711	Not included		Not included		Not included	
Duration of HAART (*per 5-years longer*)	0.924 (0.840–1.014)	0.097	Not included		Not included		Not included	
Ab anti-HCV	)							
Positive vs Negative	0.999 (0.639–1.531	0.996	Not included		Not included		Not included	
HBsAg								
Positive vs Negative	0.551 (0.258–1.072)	0.098	0.672 (0.308–1.466)	0.318	0.693 (0.298–1.468)	0.363	0.707 (0.319–1.567)	0.394
Previous AIDS diagnosis								
Yes vs No	1.427 (0.892–2.247)	0.130	1.556 (0.830–2.917)	0.168	1.544 (0.817–2.920)	0.181	1.529 (0.805–2.905)	0.195
Previous syphilis infection								
Positive vs Negative	1.536 (1.140–2.079)	0.005	1.733 (1.147–2.619)	0.009	1.643 (1.083–2.493)	0.020	1.615 (1.064–2.453)	0.025
Nadir CD4+ *(per 50-cells/μL higher)*	0.997 (0.963–1.031)	0.852	Not included		Not included		Not included	
HIV-RNA zenith	1.034 (0.912–1.178)	0.605	Not included		Not included		Not included	
*(per log*_*10*_ *copies/mL higher)*								
CD4+ *(per 50-cells/μL higher)*	0.971 (0.946–0.997)	0.029	Not included		Not included		Not included	
CD4% *(per 10% higher)*	0.961 (0.945–0.978)	<0.0001	Not included		Not included		Not included	
CD4+/CD8+ ratio (*per 0*.*1-point higher*)	0.901 (0.858–0.944)	<0.0001	0.901 (0.849–0.953)	0.0004	0.907 (0.853–0.959)	0.001	0.904 (0.851–0.957)	0.001
HIV-RNA								
≥50 vs <50 copies/mL	1.868 (0.928–3.558)	0.066	1.626 (0.978–2.703)	0.061	1.572 (0.940–2.632)	0.085	1.597 (0.951–2.681)	0.077
Number of HPV types (per 1-more type)	1.415 (1.289–1.557)	<0.0001						
≥2 vs <2	1.613 (1.183–2.193)	0.002	1.813 (1.212–2.711)	0.004	1.563 (1.031–2.368)	0.035	Not included	
HPV-16 type			Not included					
Yes vs No	1.949 (1.420–2.667)	<0.0001			1.898 (1.255–2.870)	0.002	Not included	
Number of HPV types and HPV-16 type		<0.0001	Not included		Not included			<0.001
HPV-16 –and num HPV types<2	Ref	Ref					Ref	Ref
HPV-16 –and num HPV types≥2	1.551 (1.040–2.314)	0.048					2.087 (1.239–3.517)	0.047
HPV-16 + and num HPV types<2	2.012 (1.318–3.072)	0.034					2.624 (1.521–4.529)	0.044
HPV-16 + and num HPV types≥2	2.382 (1.554–3.651)	0.020					2.589 (1.492–4.491)	0.046

Abbreviation: ART, antiretroviral therapy; MSM, men who have sex with men; HAART, highly active antiretroviral therapy; Ref, reference.

Age, HIV risk factor, years of HIV infection and other covariates with a p-value<0.20 at univariate analysis were included in the multivariate analysis

## Discussion

In our study, we found that the prevalence of abnormal cytological (ASC-US/LSIL/HSIL) was 29% with a 5.6% of HSIL in HIV-infected men who have sex with men and no clinical symptoms; these findings are generally consistent with those previously reported.

The evidence of no clinical symptoms, which may be likely explained by the fact that anal cancer and lesions are not always associated with a palpable mass, pain or bleeding, is relevant because it emphasizes the importance of patients’ follow-up, even in the absence of symptoms. In 2014 Palefsky et al [10] reported the data of 27/72 HIV-infected MSM who developed anal cancer, 14 men (52%) presented with pain, bleeding and/or a mass, but 13 of 27 (48%) men were asymptomatic at the time of presentation. Then, the authors highlighted the importance of a close follow-up to early detect invasive cancer allowing for conservative management with excision and fulguration instead of chemotherapy and radiation therapy.

Our study confirms the high prevalence of oncogenic types, already reported in other studies. The results of the ANALOGY study confirmed the high prevalence of HR-HPV among HIV infected males, and highlighted a discordance between cytology and histology with one third of the cases of AIN grade 2 or worse associated with negative cytology [11]; this possible discordance underlines the importance of screening all the MSM population.

Previous studies suggested that the high prevalence of HPV-HR in MSM could be influenced by higher risk behaviors and concomitant sexual transmitted diseases [4–6].

We also found that 31% had multiple HPV types and that half (n = 115/237, 49%) of the HPV 16 infections occurred concurrently with another HPV type. A critical issue for the assessment of multiple HPV types is the assay used for its determination. We used the Anyplex II HPV28 (H28; Seegene), a new semiquantitative real-time multiplex PCR assay for screening and genotyping 28 human papillomaviruses which was demonstrated to be analytically more sensitive, especially in the determination of non-16 and non-18 HR HPV genotypes [12]. The test exhibited greater concordance with comprehensive genotyping based on sequencing analysis and it is routinely used in our laboratory for the identification of HPV genotypes.

Given the high number of (oncogenic) HPV types in the anal canal in HIV-positive men who have sex with men, the causative HPV type involved in squamous lesions is not clear; in 2014, Richel et al. found that HPV16 was causative of intraepithelial anal lesions in <50% of cases and they concluded that anal swabs may not be useful for detecting lesion-specific HPV types [13]. Nonetheless, the association between the presence of HPV-16 type and abnormal anal cytological/histological results is well established and have been frequently shown by other cohorts [14, 15].

A previous study [6] reported that multiple HPV genotypes were associated with histological diagnosis of anal intraepithelial neoplasia (AIN) grade 3 in 76.3% of infections. Another cohort study in HIV-infected men who have sex with men by González et al. showed that multiple infections (≥2 HR-HPV genotypes) were documented in 77.7% and this factor was associated with anal SIL [16]. However, the effect of multiple HPV types in combination with HPV-16 on the risk of cytological alterations is a new finding never reported before. An interesting result is that men with multiple HPV types had a significant higher risk of an abnormal cytological finding as compared to men with single HPV infection even in the absence of with HPV 16. A recent study by Shen-Gunter et al. [17] analyzed the HPV genotypes and their proportional composition in single- and multi-infected cervical samples to explore viral diversity and characterize differences in metagenomes. They identified in LSIL/HSIL samples many HPV genotypes and the dominant(most abundant) genotypes mainly included the HPV-16 genotype; the other observed genotypes were the HPV-35 and -39.

In agreement to this study, the HPV-16 was the most frequent genotype recorded among the patients included in our study in addition to many others known to be carcinogens (i.e. HPV-18, -31, -33, -45, -51, -52, -56, -58, -59, -66). Then, we believe that the higher risk of anal SIL in patients without HPV-16 and with multiple HPV infections as compared to those with single infection may be likely explained by the fact that almost all the identified HPV genotypes, other than HPV-16, are carcinogenic and that 31% of patients evaluated in our study had multiple HPV types.

In our study, we also found that a lower CD4+/CD8+ ratio, a detectable viral load and a previous diagnosis of syphilis are associated with a higher risk of having an abnormal cytological finding; subjects with a previous AIDS diagnosis also tended to have a higher risk of cytological abnormalities. In the ICONA cohort, the CD4+/CD8+ ratio, in particular lower than 0.3, was found to be an independent risk factor for the development of non-AIDS related diseases and death [18]. Over a total of 14,926 PYFU, clinical progression occurred in 336 patients; 71 had non-AIDS defining malignancy, with a median value of last ratio of 0.56 (0.31-0-79). In our study, the median CD4+/CD8+ ratio in patients with SIL was 0.62 (0.44–0.93).

With respect to the diagnosis of AIDS, previous data showed that it is a risk factor for SIL [4, 17]; Goedert and colleagues [19], reported that the relative risk of HSIL development is 31.7 after the diagnosis of AIDS.

Syphilis is related to high risk sexual behaviors and it is highly prevalent in HIV infection [20]. In a Chinese study [21], syphilis was detected in 55.8% of patients with HIV and HPV coinfection, confirming that the presence of a sexually transmitted disease could induce another infection.

We considered in Model 4, 5 and 6 the risk of having abnormal cytological findings, including ASC-US. We calculated these models because it is known that in HIV-infected women there is a higher risk of SIL following the appearance of ASC-US, especially in those with low CD4 count. No clear data are available in men; in a prospective study, Schofield et al. found a high prevalence of false negative citology and, then, suggested to strictly monitor men who have sex with men with any cytological abnormality [11]. Similarly, in the setting of HIV-positive men, we suggest a close monitoring of patients with ASC-US.

This study has some limitations. First, we did not collect the number of partners, that is known to be a risk factor for anal squamous intraepithelial lesions [22]. Secondly, our results may be almost generalized to MSM on treatment with ART as the subjects who performed the HPV screening were mainly MSM (80%) and on treatment subjects (98%).

In conclusion, in HIV-1 infected males who have sex with males, the concomitant presence of HPV-16 and multiple HR genotypes was associated with an increased risk of abnormal cytological findings in addition to a lower CD4+/CD8+ ratio, a positive viral load and with a previous diagnosis of syphilis.

These data highlight the importance of screening multiple HPV genotypes in HIV-infected patients.

## Supporting information

S1 Database File contain analyzed data (IDD-HSR) for this paper. (PDF)
