# Supplementary material for: Presence of multiple genotypes in subjects with HPV-16 infection is highly associated with anal squamous intraepithelial lesions in HIV-1 infected males
Source: PLoS One. 2017 Oct 31;12(10):e0186367. doi: 10.1371/journal.pone.0186367 (PMC5663371; doi:10.1371/journal.pone.0186367)
Supplement: S1 Database — (PDF) [file pone.0186367.s001.pdf]

ID	NASCITA	ESAME	SESSO	EPIDEMIOLOGIA	D_AIDS	cdc	D_ARV	D_HIV	ZENITH	NADIR	HCVAB	HBSAG	SIFILIDE	DROPOUT	HPV11	HPV16	HPV18	HPV6_11	HPV_31	HPV_33	HPV_35	HPV_39	HPV_45	HPV_51	HPV_52	HPV_56	HPV_58	HPV_59	HPV_68	HPV_73	HPV_HR	HPV_HR_OVERALL	ART	CD4	CD4p	CD4_CD8	HIVRNA	AIN	VACCINAZIONE	D_VACCINAZIONE	CITOLOGICO	ISTOLOGICO
4	02/03/1964	15/09/2011	M	4	01-dic-94	C3	01/10/1990	01/01/1984	5100	87	1	0	9	0		0	0														1	1	1	182	10,7		0,9	0	0		0	
6	02/10/1963	14/10/2010	M	1		B2	02/04/1997	01/01/1989	41000	219	0	9	1	0		0	0														0	0	1	924	28		0,9	0	0		0	
21	04/06/1966	07/01/2010	M	3		B3	01/01/1990	01/02/1987	1500000	74	0	0	0	0		0	0														0	0	1	442	17	0,33	36	0	0		0	
30	01/11/1960	06/09/2007	M	1	20-giu-94	C3	24/02/1992	01/01/1991	166657	62	0	0	0	0																	1	1	1	65	7,2	0,11	18474	1	0			
37	22/11/1955	25/06/2009	M	5	17-lug-92	C3	27/05/1992	01/03/1992	220000	188	0	1	0	0																	0	0	1	726	26,9	0,62	0,9	0	0			
63	27/11/1954	15/11/2007	M	1	16-giu-94	C3	16/05/1990	01/01/1987	2400	16	0	0	1	0																	1	1	1	1153	37,2		49	0	0			
89	09/06/1967	06/08/2014	M	2		A3	01/01/1989	01/01/1984	46373	153	1	0	0	0		0	0														1	1	1	770	29,6		0,9	0	0		0	
121	02/09/1952	13/02/2014	M	1		C3	10/04/1992	01/04/1987	459312	36	0	0	1	0		0	0														1	1	1	554	19,1	0,44	0,9	0	0		0	
139	26/08/1958	17/02/2011	M	1		A3	04/11/1997	01/10/1992	135900	45	0	9	0	0		0	0														0	0	1	650	22,4	0,42	4761	1	0		1	
177	20/03/1964	27/03/2014	M	1	01-lug-95	C3	17/07/1995	01/01/1992	120000	358	0	0	1	0		0	1														1	1	1	702	31,9		0,9	1	0		1	
223	06/03/1961	15/11/2007	M	3		B2	11/09/1995	01/01/1992	140000	329	1	9	1	0																	1	1	1	752	13,2	0,23	4350	0	0			
249	17/09/1970	05/03/2009	M	1		A2	06/09/2004	31/03/1993	27636	334	0	9	0	0																	0	0	1	1099	33,3		49	0	0			
252	10/07/1955	18/10/2007	M	1	01-nov-94	C3	21/03/1993	01/01/1992	466100	137	1	9	0	0																	1	1	1	241	14,2	0,2	466100	1	0			
252	10/07/1955	22/06/2010	M	1	01-nov-94	C3	21/03/1993	01/01/1992	466100	137	1	9	0	0		0	0	0	0	0	0	0	0	0	0	0	0	0	0	0	0	0	1	293	15,4		92830	0	0		0	
254	05/11/1958	07/01/2016	M	1			15/06/1996	01/01/1993	82000	276	0	0	0	0	1	0	0	0		0	0	0	0	0	0	0	0	0	1	0	1	1									0	
273	19/03/1951	11/02/2010	M	1		A3	08/03/1993	01/01/1986	224412	131	0	0	1	0																	0	0	1	542	27,1	0,63	0,9	0	0			
301	31/08/1964	21/06/2007	M	3	01-gen-93	C3	02/10/1992	01/01/1989	473000	2	0	0	0	0																	1	1	1	684	22,8	0,51	49	2	0			
309	28/09/1955	07/05/2009	M	1		A2	16/03/1998	01/12/1992	24346	317	0	0	1	0																	1	1	1	510	17	0,24	49	0	0			
331	10/10/1948	15/05/2008	M	2		B2	24/02/1992	01/01/1989	34000	451	1	1	0	0																	0	0	1	1553	31,7		49	0	0			
368	20/02/1942	17/06/2010	M	1	01-ott-96	C3	11/01/1993	01/01/1986	1200000	75	9	9	1	0		0	0														1	1	1	571	16,3		0,9	0	0		0	
426	11/08/1963	03/06/2010	M	1		A2	17/02/1992	01/05/1991	230000	384	0	0	1	0		1	0														1	1	1	764	38,2		0,9	0	0		0	
452	21/06/1961	06/08/2009	M	1		B3	17/11/2008	01/01/1992	117130	151	0	1	1	1																	1	1	1	426	22,4	0,53	0,9	1	0			
470	14/02/1956	31/05/2007	M	1		C2	10/05/1993	24/01/1990	28000	462	0	9	1	0																	0	0	1	1312	32		49	0	0			
564	28/06/1964	10/12/2010	M	1		A3	01/01/2004	01/01/2004	41620	104	0	9	0	0		0	1														1	1	1	193	8,4	0,13	40	1	0		1	
565	04/01/1970	16/11/2011	M	1		B2	10/08/1992	01/01/1989	6825	203	0	9	0	0		1	0	1	0	0	1	0	1	0	0	0	0	0	0	0	1	1	1	509	15,9		0,9	3	0		3	3
573	17/01/1963	02/08/2012	M	1		C3	24/09/1993	01/01/1985	930000	175	0	0	0	0		0	0														1	1	1	1334	29	0,88	36	0	0		0	
599	08/08/1960	21/10/2010	M	4	26-giu-97	C3	01/09/1992	01/01/1985	5569	313	1	1	0	0		0	0														0	0	1	574	31,9	0,65	36	0	0		0	
649	17/08/1961	21/05/2009	M	1	01-gen-99	C3	16/07/1992	01/01/1986	44000	153	0	1	0	0																	1	1	1	458	21,8		0,9	0	0			
669	05/09/1963	24/11/2011	M	1		B2	05/12/1996	01/01/1992	310000	456	0	9	1	0		0	0														0	0	1	460	24,2		20350	0	0		0	
729	10/10/1957	29/11/2007	M	1		B2	05/02/1990	01/01/1987	31694	308	0	0	0	0																	1	1	1	718	29,9	0,82	49	1	0			
767	22/02/1954	10/07/2014	M	1		A2	24/04/1996	01/12/1992	240168	496	0	0	0	0		0	0	0	0	0	0	0	1	0	0	0	0	0	1	0	1	1	1	1395	27,8		0,9	0	0		0	
829	10/03/1951	28/05/2015	M	1			31/10/1997	16/09/1985	15000	339	0	0	1	0	0	0	0	0	1	0	0	0	0	0	1	0	0	0	0	0	1	1	1	1086	47,2	1,42	0,9	0	0		0	
832	19/04/1954	09/05/2013	M	1	20-apr-11	C3	24/11/1998	01/01/1994	63352	207	0	0	0	0		0	0														0	0	1	529	19,6	0,33	0,9	0	0		0	
990	14/02/1959	26/07/2016	M	5			01/05/1997	01/02/1993	300000	490	0	0	0	0	0	1	0	0		0	0	0	0	0	0	0	0	0	0	0	1	1		991	36,7	0,96	0,9				0	
1027	02/05/1963	03/02/2011	M	1	01-giu-94	C3	01/01/1995	28/06/1994	550000	8	0	0	1	0		1	1														1	1	1	155	8,6		36	1	0		2	
[illegible]	[illegible]	[illegible]	[illegible]	[illegible]	[illegible]	[illegible]	[illegible]	[illegible]	[illegible]	[illegible]	[illegible]	[illegible]	[illegible]	[illegible]	[illegible]	[illegible]	[illegible]	[illegible]	[illegible]	[illegible]	[illegible]	[illegible]	[illegible]	[illegible]	[illegible]	[illegible]	[illegible]	[illegible]	[illegible]	[illegible]	[illegible]	[illegible]	[illegible]	[illegible]	[illegible]	[illegible]	[illegible]	[illegible]	[illegible]	[illegible]	[illegible]	[illegible]

6194	24/05/1968	04/02/2016	M	1			19/12/2013	06/12/2005	71670	222	1	0	1	0	0	0	0
6195	15/03/1968	25/11/2010	M	1		A2	14/06/2006	20/01/2006	111838	277	1	0	1	0		0	0
6201	18/11/1970	30/09/2010	M	5		B1	17/06/1996	01/01/1993	49	513	0	0	0	0		1	1
6205	18/01/1980	10/11/2011	M	5		A2	03/12/2012	01/01/2004	61390	416	0	0	1	0		0	0
6238	17/01/1975	14/01/2010	M	1		A2	13/03/2012	04/11/2004	123300	414	0	9	1	0			
6239	04/06/1971	17/12/2009	M	1		A1	01/09/1998	07/04/1995	31230	595	0	9	0	0			
6252	13/01/1970	26/08/2010	M	5		A2	15/06/1997	01/01/1997	49	378	0	9	1	0		0	0
6265	15/05/1986	19/12/2013	M	1		A3	17/06/2005	01/01/2004	6355	166	0	9	1	0		0	0
6279	16/04/1960	08/08/2013	M	2			15/12/1995	01/01/1986	36	356	9	0	0	0		0	0
6281	20/11/1956	21/04/2016	M	1			10/09/1997	01/05/1993	53770	352	0	0	1	0	1	0	0
6284	02/05/1972	04/07/2013	M	1		A2	03/11/2009	13/09/2004	39296	294	0	1	1	0		1	0
6285	19/12/1970	12/04/2012	M	1		A2	01/08/2006	01/02/2006	56754	285	0	0	1	0		0	0
6299	08/02/1959	17/11/2011	M	4		C3	27/01/2006	01/01/1982	84170	66	0	0	0	0		0	1
6308	10/06/1953	03/01/2013	M	1	20-apr-06	C3	10/04/2006	04/04/2006	137022	216	0	0	9	0		0	0
6315	26/10/1968	28/04/2011	M	5		A1	15/09/2003	05/04/2002	101	565	9	9	0	0		1	0
6318	15/04/1970	29/01/2015	M	1			07/02/2005	01/04/2004	191	470	0	0	1	0	0	0	0
6324	28/07/1969	06/11/2008	M	1		B1	14/12/2005	05/12/2005	128897	500	0	9	1	0			
6328	20/04/1947	07/04/2016	M	5			01/01/1995	01/01/1994	21156	418	0	9	1	0	0	0	0
6357	17/11/1971	01/12/2011	M	5		A1	01/07/2001	01/03/2001	47500	518	0	9	1	0		0	0
6361	17/02/1966	09/06/2016	M	5					112	668	0	9	1	0	0	0	0
6361	17/02/1966	22/11/2007	M	1		A1		01/01/1985	112	721	0	9	1	0			
6364	01/01/1962	14/05/2009	M	5		A2	01/12/1995	01/11/1992	30942	359	0	0	1	0			
6364	01/01/1962	15/05/2014	M	5		A2	01/12/1995	01/11/1992	30942	295	0	0	1	0		1	0
6377	08/05/1974	03/12/2009	M	5		A3	27/07/2004	05/07/2004	2260540	120	0	9	1	0			
6395	06/02/1961	13/11/2014	M	1			01/08/2005	01/06/2004	978	211	0	0	0	0	0	0	0
6414	14/10/1979	22/04/2010	M	1		A2	05/02/2005	01/08/2004	178	483	9	9	1	0		1	0
6416	29/11/1976	28/03/2013	M	1		A1		20/02/2013	49	870	0	9	0	0		0	0
6436	02/10/1976	21/04/2010	M	1		A1	01/04/2005	21/04/2005	2125	1660	0	9	0	0		0	0
6459	28/08/1969	31/03/2016	M	1	01-gen-96	C3	30/06/1992	01/01/1992	28085	203	1	0	1	0	0	1	0
6460	02/04/1963	13/10/2011	M	1		A2	01/01/1999	01/01/1995	192833	430	0	0	0	0		1	0
6479	04/12/1960	01/12/2011	M	5		A1	15/03/1998	01/03/1986	49	611	9	9	9	0		1	0
6479	04/12/1960	07/02/2013	M	5		A1	15/03/1998	01/03/1986	49	611	9	9	9	0		0	0
6494	10/12/1975	30/04/2015	M	1			20/07/2005	01/01/2005	141506	298	0	1	1	0	0		0
6512	11/06/1968	31/01/2008	M	1		A2	05/09/2006	26/05/2006	246124	250	0	9	1	0			
6514	22/03/1971	08/05/2014	M	1		A3	17/07/2008	30/06/2005	487872	191	0	0	0	0		0	0
6516	19/08/1974	12/07/2016	M	1			23/03/2006	01/01/2006	163917	283	0	0	1	0	0	0	0
6517	02/04/1968	10/04/2014	M	1		A2	02/04/2009	03/03/2006	15172	322	1	0	1	0		0	1
6518	22/01/1963	16/06/2011	M	1		A2	19/11/2007	01/01/2005	141768	257	0	9	0	0		1	1
6525	10/04/1969	22/07/2010	M	1		A2	01/03/2006	17/02/2006	427433	280	0	0	1	0		0	0
6525	10/04/1969	14/03/2013	M	1		A2	01/03/2006	17/02/2006	427433	280	0	0	1	0		0	0
6539	30/07/1976	22/11/2007	M	1		A3	27/09/2006	13/06/2006	270000	72	1	0	1	0			
6548	03/04/1959	04/11/2010	M	1	26-set-14	C3	18/01/2008	01/06/2006	400857	253	0	0	1	0		0	1

[illegible]

9953	30/07/1989	07/05/2015	M	1			21/07/2014	26/03/2014	10003	747	0	0	1	0	1	1	0	1	0	0	0	1	0	0	0	1	0	0	0	0	1	0	0	0	1	1	1	1479	34,4	0,98	0,9	0	1	01/10/2014	0	
9964	09/05/1985	30/06/2016	M	1			29/05/2014	13/03/2014	106176	268	0	0	0	0	0	0	0	1		0	1	1		0	1	0	0	0	0	1	0	0	0	0	1	1		554	15,4	0,29	0,9				1	
9965	15/09/1989	05/03/2015	M	1			22/05/2014	07/03/2014	755355	177	0	0	1	0		0	0																		0	0	1	446	34,3	1,19	0,9	0	0		0	
9969	25/04/1987	18/12/2014	M	1			13/05/2014	08/04/2014	88221	495	0	0	0	0	0	1	0	0	0	0	0	0	0	0	0	0	0	0	0	0	1	0	0	0	1	1	1	946	39,4	0,82	0,9	0	0		0	
9981	13/02/1984	07/11/2014	M	1			05/03/2014	08/08/2013	39	204	0	0	1	0	0	0	0	0	1	0	1	0	1	0	1	0	1	1	0	0	0	0	0	0	1	1	1	490	30,6	0,53	39	0	0		0	
9985	19/05/1976	26/06/2014	M	1		A3	23/05/2014	02/05/2014	1080127	59	0	0	0	0		0	0	0	0	0	1	0	0	0	1	0	0	0	0	0	0	0	1	0	1	1	1	407	33,9	0,83	1031	1	0		2	
10005	22/04/1973	19/05/2016	M	1			04/09/2014	22/05/2014	58164	448	0	0	1	0	1	0	0	0		0	0	0		0	0	0	0	0	0	0	0	0	0	0	1	1		954	29,8	0,67	0,9				0	
10008	14/07/1961	05/04/2016	M	1			13/06/2014	06/06/2014	5196	823	0	1	0	0	0	0	0	0		1	0	0		1	0	0	0	0	0	0	0	0	0	0	1	1		1319	47,1	1,61	0,9				0	
10011	07/04/1985	17/12/2015	M	1			23/06/2014	06/06/2014	6078	260	0	0	0	0	0	0	0	0	0	1	1	0	0	1	1	0	0	0	0	0	0	0	0	0	1	1	1	955	39,8	1,1	0,9	0	0		0	
10016	26/05/1987	01/10/2015	M	1			01/02/2008	01/05/2008	0,9	746	0	0	0	0	0		0	0	1	0	0	0	1	0	0	0	0	0	0	0	0	0	0	0	1	1	1	746	43,9	1,61	0,9	0	0		0	
10021	06/03/1987	10/03/2016	M	1			07/08/2014	10/06/2014	1462952	382	0	0	0	0	0	0	0	0		0	1	0		0	1	0	0	0	0	0	1	0	0	0	1	1		748	37,4	1,03	0,9				0	2
10023	07/09/1981	23/06/2016	M	1			04/09/2014	01/02/2014	27005	298	0	0	0	0	0	1	0	0		0	0	0		0	0	0	0	0	0	1	0	0	0	0	1	1		633	33,3	0,86	39				0	
10032	17/12/1965	25/06/2015	M	1			10/07/2014	25/06/2014	38361	465	0	0	0	0	0	0	1	0	0	0	0	0	0	0	0	0	0	0	1	0	1	0	0	0	1	1	1	739	33,6	0,93	0,9	0	0		0	
10036	20/08/1953	18/02/2016	M	1			27/08/2014	27/06/2014	4344	312	0	0	0	0	0	1	0	0		0	0	0		0	0	0	0	0	0	0	0	0	0	0	1	1		726	26,9	1,09	0,9				0	
10045	01/11/1988	01/10/2015	M	1			06/10/2014	20/06/2014	16438	969	0	0	0	0	0	0	0	1		1	0	1		1	0	0	0	0	0		1	0	0	0	1	1	1	1149	35,9	0,88	0,9	0	0		0	
10051	21/04/1974	09/07/2015	M	1			07/08/2014	01/08/2014	1995169	431	1	0	1	0	0	0	0	0	0	0	0	0	0	0	0	0	0	0	0		1	0	0	0	1	1	1	1044	36	0,9	0,9	1	0		1	
10057	19/08/1975	26/05/2016	M	1			01/03/2008	01/08/2006	39	853	0	0	1	0	1	1	0	0		0	1	0		0	1	0	1	0	0	0	1	0	0	0	1	1		983	35,1		0,9				0	
10060	20/05/1977	10/09/2015	M	1			01/09/2014	28/08/2014	1000001	545	0	9	1	0	0	1	0	0	0	0	1	0	0	0	1	0	1	1	0	0	1	0	0	1	1	1	1	1512	33,6	0,82	39	0	0		0	
10061	09/07/1976	27/11/2014	M	1			24/02/2009	19/09/2005	0,9	590	0	9	0	0	1	0	0	0	0	0	0	0	0	0	0	0	0	0	0	0	1	0	0	0	1	1	1	590	26,8	0,77	0,9	0	0		0	
10064	19/07/1983	09/07/2015	M	1			10/10/2014	22/08/2014	358398	698	0	0	1	0	0	0	0	0	0	1	1	0	0	1	1	0	0	1	0	0	1	0	1	0	1	1	1	1033	41,3		0,9	0	0		0	
10071	05/03/1954	19/03/2015	M	2			23/12/1997	15/12/1999	39	288	0	9	1	0	0	0	1	0	0	0	0	0	0	0	0	0	0	0	0	1	0	0	1	0	1	1	1	288	16		0,9	1	0		2	
10073	05/11/1990	19/05/2016	M	1			30/09/2014	06/09/2014	11565	338	0	1	0	0	0	0	0	0		0	0	0		0	0	1	0	0	0	0	0	0	0	0	1	1		751	25,9	0,71	0,9				0	
10074	13/02/1975	15/10/2015	M	1			27/08/2014	08/08/2014	2965	475	0	0	1	0	0	1	0	0	1	0	0	0	1	0	0	0	0	0	1	0	1	0	1	0	1	1	1				0,9	0	0		0	
10085	10/10/1975	18/06/2015	M	1			01/11/2009	01/07/2009	0,9	1105	0	0	0	0	0	0	0	0	0	0	0	0	0	0	0	0	0	0	0	0	0	0	0	0	0	0	1	1359	45,3	1,3	0,9	0	0		0	
10089	17/02/1988	14/04/2016	M	1			26/11/2014	01/04/2011	1072	433	0	0	0	0	0	0	1	0		0	0	0		0	0	0	0	0	0	0	0	0	0	0	1	1		624	39	0,79	0,9					1
10106	15/07/1972	03/12/2015	M	5			22/10/2014	15/10/2014	217118	245	0	0	1	0	0	0	0	0	0	0	1	0	0	0	1	0	0	0	0	0	1	0	0	0	1	1	1	549	28,9		39	0	0		0	
10108	30/11/1979	03/09/2015	M	1			04/12/2014	20/10/2014	38874	386	0	0	1	0	0	1	0	0	0	0	1	0	0	0	1		0	1	0	0	1	0	0	0	1	1	1	581	24,2	0,46	0,9	0	0		0	
10124	16/06/1986	12/11/2015	M	1				10/10/2006	10349	796	0	0	0	0	0	0	1	0	0	1	0	0	0	1	0	0	0	1	0	0	1	0	0	0	1	1	0	884	22,1	0,45	10349	0	0		0	
10140	05/07/1990	04/06/2015	M	1			28/01/2015	13/11/2014	1132	694	0	0	0	0	0	0	0	0	0	0	0	0	0	0	0	1	0	0	0	0	1	0	1	0	1	1	1	694	36,5	0,88	0,9	0	0		0	
10158	12/05/1963	11/06/2015	M	1			09/06/2014	08/05/2014	82	651	0	0	0	0	0	0	0	0	0	0	0	0	0	0	0	1	1		1	0	0	0		0	1	1	1	680	35,8	0,89	0,9	0	0		0	
10178	18/09/1985	26/05/2016	M	1			07/01/2015	20/11/2014	151012	752	0	0	1	0	0	1	0	0		0	1	0		0	1	0	0	0	0	0	1	1	0	1	1	1		817	35,5	0,83	0,9				0	
10208	06/06/1985	25/06/2015	M	1			17/12/2014	09/12/2014	100217	574	0	0	1	0	1	1	1	1	0	0	0	1	0	0	0	1	0	0	0	0	1	0	0	0	1	1	1	774	35,2	0,88	0,9	0	0		0	
10214	22/11/1974	12/11/2015	M	1			22/12/2014	23/11/2014	14705	680	0	0	0	0	0	1	0	0	0	0	0	0	0	0	0	0	0	0	0	0	1	0	0	0	1	1	1	826	48,6	1,61	0,9	0	0		0	
10257	06/08/1974	10/05/2016	M	1			17/02/2015	09/12/2014	81912	158	0	0	1	0	0	1	0	1		1	0	1		1	0	0	0	0	0	0	0	0	1	0	1	1		216	19,6	0,36	0,9				0	
10271	06/04/1983	18/06/2015	M	1			08/01/2015	15/12/2014	12802	608	0	0	1	0	0	0	1	1	0		0	1	0		0	0	1	0	0	0		0	0		1	1	1	911	41,4	1,11	0,9	0	0		0	
10278	04/11/1967	28/04/2016	M	1			31/12/2014	23/12/2014	29198	430	9	0	1	0	0	0	1	0		0	0	0		0	0	0	0	0	0	0	0	0	1	1	1	1		867	29,9	0,86	0,9				1	
10292	12/09/1979	25/06/2015	M	1			09/06/2007	01/01/2001	0,9	517	0	9	0	0	0	0	0	0	0	0	0	0	0	0	0	0	0	0	0	0	1	0	0	0	1	1	1	657	36,5	1,06	0,9	0	0		0	
10299	03/01/1990	31/03/2016	M	1			09/01/2015	30/12/2014	141422	148	0	9	1	0	0	0	0	0		0	0	0		0	0	0	0	0	0	0	0	0	1	0	1	1		541	20,8	0,43	0,9				0	
10338	13/05/1985	28/04/2016	M	1			01/07/2015	04/09/2009	70956	1150	0	9	1	0	1	1	1	0		0	1	0		0	1	1	0	0	1	0	0	0	0	1	1	1		1474	28,9	0,55	0,9				1	
10339	14/10/1982	05/04/2016	M	1			24/02/2015	09/12/2014	7255	335	0	0	1	0	1	0	0	0		0	1	0		0	1	0	1	0	0	0	1	0	1	0	1	1		600	31,6	0,74	0,9				2	1
10351	19/06/1981	19/04/2016	M	1			10/03/2015	20/01/2015	1951	396	0	0	0	0	0	0	1	0		0	0	0		0	0	0	0	1	0	1	0	0	0	0	1	1		854	26,7	0,55	0,9				0	
10362	16/08/1986	21/07/2016	M	1			04/01/2016	20/01/2015	29684	696	0	0	0	0	0	0	0	0		0	0	0		0	0	0	1	0	0	0	1	0	0	0	1	1		921	27,1	0,62	39				0	
10363	05/05/1961	04/02/2016	M	1			27/07/2015	19/01/2015	8617	979	0	0	1	0	0	1	0	1		1	0	1		1	0	1	0	0	0	0	1	0	0	0	1	1		1119	33,9	1,07	0,9				2	
10369	25/03/1968	03/03/2016	M	1			17/02/2015	09/02/2015	7560	267	0	0	1	0	0	0	0	1		0	0	1		0	0	0	0	0	0	0	0	0	0	1	1	1		363	33	1,22	0,9				0	
10376	22/10/1969	04/06/2015	M	1			01/07/2013	01/01/2013	0,9	352	0	9	0	0	0	1	0	0	0	0	0	0	0	0	0	0	0	0	1	0	0	0	1	0	1	1	1	352	17,6			0	0		0	
10382	30/07/1981	20/08/2015	M	1			13/04/2015	20/02/2015	12472	787	0	0	0	0	1	0	0	1	1	0	0	1	1	0	0	0	1	0	0	0	1	0	0	0	1	1	1	1201	42,9	1,43	39	1	0		2	3
10382	30/07/1981	21/06/2016	M	1			13/04/2015	20/02/2015	12472	787	0	0	0	0	1	0	0	1		0	0	1		0	0	0	1	0	0	0	1	0	0	0	1	1		1291	46,1	1,61	0,9				0	2
10387	02/10/1982	21/05/2015	M	1			10/04/2015	01/10/2013	3193054	169	0	0	0	0	0	0	0	0	0	0	1	0	0	0	1	0	1	0	0	0	0	1	0	0	1	1	1				0,9	0	0		0	
10387	02/10/1982	21/07/2016	M	1			10/04/2015	01/10/2013	3193054	169	0	0	0	0	0	0	0	0		0	0	0		0	0	0	0	0	0	0	0	0	0	0	0	0		456	16,3	0,28	39				0	
10390	13/02/1969	19/05/2016	M	1			14/04/2015	23/02/2015	83581	372	0	0	0	0	0	0	0	1		0	0	1		0	0	0	0	0	0	0	0	0	0	0	0	1		624	31,2	0,83	0,9				0	
10405	16/04/1993	30/07/2015	M	1			17/04/2015	07/04/2015	23103	398	0	0	0	0	0	0	1	0	1	0	0	0	1	0	0	1	0	0	0	0	0	0	0	0	1	1	1	643	25,7	0,54	0,9	0	0		0	
10408	18/02/1975	03/03/2016	M	1			01/12/2012	01/11/2012	0,9	678	0	0	0	0	0	0	0	0		0	0	0		0	0	0	0	1	0	0	0	0	0	0	1	1		678	24,2	0,53	0,9				0	
10409	24/12/1971	03/03/2016	M	1			01/12/2012	01/12/2012	0,9	720	0	0	0	0	0	0	0	1		1	0	1		1	0	0	0	0	0	0	0	0	0	0	1	1		876	30,2	0,98	0,9				0	
10417	29/04/1966	10/03/2016	M	1			27/04/2015	26/03/2015	175186	228	0	0	0	0	0	1	0	0		0	0	0		0	0	0	0	0	0	0	0	0	1	1	1	1		530	24,1	0,58	0,9					1
10423	09/08/1986	03/05/2016	M	1			14/05/2015	24/04/2015	132712	544	0	0	0	0	0	0	1	1		0	0	1		0	0	0	0	0	1	0	0	0	0	1	1	1		1417	38,3	0,91	0,9				1	
10436	20/01/1978	05/05/2016	M	1			10/07/2015	12/05/2015	6688	447	0	0	0	0	1	0	0	0		0	0	0		0	0	0	0	1	0	0	0	0	1	0	1	1		662	28,8	0,68	0,9				0	
10441	21/11/1988	18/02/2016	M	1			05/03/2016	14/05/2014	11087	578	0	0	1	0	1	0	0	1		0	0	1		0	0	0	0	0	0	0	0	0	0	0	1	1		699	25,9		11087				0	
10442	24/05/1964	11/08/2016	M	1	15-gen-14	C3	26/02/2014	15/02/2014	0,9	279	1	0	1	0	0	1	0	1		1	1	1		1	1	0	1	0	0	0	0	1	1	0	1	1		483	28,4	0,72	0,9				0	
10449	28/03/1984	10/05/2016	M	1			14/07/2015	14/05/2015	11648	382	1	0	1	0	0	1	0	0		0	0	0		0	0	1	0	0	0	0	0	0	1	1	1	1		771	26,6	0,55	0,9				2	
10453	10/08/1976	07/01/2016	M	1			04/01/2010	15/12/2009	0,9	828	0	0	0	0	1	0	0	0		0	1	0		0	1	0	0	0	0	0	1	0	0	0	1	1		876	29,2	0,96	0,9				0	
10456	03/08/1992	26/04/2016	M	1			22/06/2015	10/06/2015	775024	69	0	0	0	0	0	0	0	0		0	0	0		0	0	1	0	0	0	0	0	0	0	0	1	1		204	18,5	0,3	0,9				0	
10456	03/08/1992	29/07/2016	M	1			22/06/2015	10/06/2015	775024	69	0	0	0	0	0	0	0	0		0	0	0		0	0	0	0	0	0	0	0	0	0	0	1	1		328	20,5	0,4	0,9				0	0
10468	03/09/1979	26/05/2016	M	1			23/06/2015	18/06/2015	209	312	0	0	1	0	0	0	0	0		0	0	0		0	0	0	1	0	0	0	0	0	0	0	1	1		1426	32,4	0,68	0,9				0	
10470	05/01/1974	23/06/2016	M	1			05/08/2015	17/06/2015	1115	405	0	0	0	0	1	0	0	0		1	0	0		1	0	0	0	0	0	0	0	1	1	1	1	1		591	21,1	0,87	0,9				0	
10486	22/08/1972	19/04/2016	M	1			03/08/2015	22/07/2015	1394828	262	0	0	0	0	0	0	0	0		0	0	0		0	0	0	0	0	0	0	1	0	0	0	1	1		415	31,9	0,74	115				0	
10492	25/10/1976	17/03/2016	M	1			05/08/2015	21/07/2015	468960	12	0	0	0	0	0	0	0	0		1	1	0		1	1	0	0	0	0	0	0	1	0	0	1	1		206	8,6	0,15	0,9				1	
10494	04/12/1972	18/09/2015	M	1			23/11/2015	22/07/2015	10750	454	0	0	0	0	1	0	0	0	0	0	0	0	0	0	0	0	0	0	0	0	0	0	0	0	0	1	1	787	25,4		0,9	1	0		2	
10513	05/05/1988	28/06/2016	M	1			23/10/2015	10/09/2015	46548	532	0	0	1	0	0	0	0	0		0	0	0		0	0	0	0	0	0	0	0	0	0	0	1	1		708	24,4	0,57	0,9				0	
10526	15/01/1990	03/03/2016	M	1			20/11/2015	25/09/2015	116132	466	0	0	0	0	1	1	1	0		0	1	0		0	1	0	0	0	0	0	0	1	0	1	1	1		615	32,5	0,71	54				0	
10536	18/06/1980	23/06/2016	M	1			24/11/2015	01/10/2015	7092	903	1	0	1	0	0	0	0	0		0	0	0		0	0	0	1	0	0	0	0	0	0	0	1	1		1221	35,9	0,8	0,9				0	
10537	02/11/1988	10/05/2016	M	5			13/02/2013		39	891	1	9	1	0	0	0	0	0		0	0	0		0	0	0	0	0	0	0	0	0	0	0	1	1		1072	33,5	0,6	0,9				0	
10541	18/04/1972	28/04/2016	M	1			28/10/2015	01/10/2015	114631	288	0	0	1	0	1	0	0	0		0	0	0		0	0	1	0	0	0	0	0	0	0	0	1	1		710	33,8	1,26	0,9				0	
10551	17/02/1990	31/05/2016	M	1			16/11/2015	26/10/2015	382063	197	0	0	0	0	0	1	0	0		0	1	0		0	1	0	0	0	0	0	1	0	0	0	1	1		482	24,1	0,69	0,9				0	
10562	28/07/1962	26/11/2015	M	1			01/07/2005	01/06/2005	0,9	827	0	9	1	0	1	0	0	0	0	0	0	0	0	0	0	0	0	0	0	0	0	0	0	0	1	1	1	827	28,5	0,52	0,9	0	0		0	
10564	11/04/1968	25/08/2016	M	1			30/10/2015	26/10/2015	0,9	927	0	0	1	0	0	0	0	1		0	0	1		0	0	0	0	1	0	0	0	0	0	0	1	1		1169	28,5	0,56	0,9				1	
10571	27/10/1977	23/08/2016	M	1			20/12/2011	30/11/2011	0,9	1195	0	0	1	0	1	0	0	1		0	0	1		0	0	0	0	0	0	0	1	0	0	0	1	1		1195	27,8	0,72	0,9				2	
10587	22/04/1982	21/06/2016	M	1			01/05/2010	01/01/2002		955	0	0	1	0	0	0	0	0		0	0	0		0	0	0	0	0	0	1	0	0	1	0	1	1		1128	37,6	1,13	0,9				0	
10596	05/05/1994	29/03/2016	M	1			15/03/2016	27/04/2013		768	0	0	1	0	0	1	0	0		0	0	0		0	0	0	0	0	0	0	1	1	0	0	1	1		785	30,9	0,9	415				0	
10605	17/08/1988	10/03/2016	M	1			03/03/2016	19/01/2016		538	0	0	1	0	0	0	0	1		1	0	1		1	0	1	0	0	1	1	1	0	0	1	1	1		538	26,9	0,54	18191				0	
10609	20/02/1968	28/04/2016	M	5			01/01/1993	01/01/1990		6	9	9	0	0	0	0	0	0		0	0	0		0	0	0	0	0	0	0	0	0	0	0	0	0		20	1,6	0,02	15584				0	
10611	06/10/1972	26/05/2016	M	5	01-giu-10	C3	01/06/2010			598	0	0	1	0	1	0	0	0		0	0	0		0	0	0	0	0	0	0	0	0	0	0	0	1		598	27,2	0,71	0,9				0	
10622	23/01/1985	16/06/2016	M	1			04/03/2016	29/02/2016		409	1	0	0	0	0	0	0	0		0	0	0		0	0	0	0	0	0	0	0	0	0	0	0	0		690	23	0,48	0,9				0	
10623	03/07/1979	17/05/2016	M	1			06/05/2016	23/02/2016		364	0	0	0	0	0	0	0	0		1	0	0		1	0	0	0	0	0	0	0	0	0	0	1	1		389	26,4	0,54	64000				0	
10625	27/04/1989	02/08/2016	M	1			31/05/2016	22/02/2016		496	0	0	0	0	0	0	0	0		0	0	0		0	0	0	0	0	0	0	0	0	0	0	1	1		563	29	0,36	19				2	
10641	12/07/1988	03/06/2016	M	1			26/04/2016	03/02/2016		707	0	0	0	0	1	0	0	1		1	0	1		1	0	0	0	0	0	0	0	0	0	0	1	1		1333	32,5	1,03	751					
10651	14/01/1991	26/04/2016	M	1			23/03/2016	01/03/2016		359	0	0	0	0	0	0	0	0		0	0	0		0	0	0	0	0	0	0	0	0	0	0	1	1		359	23,9	0,44	5515				0	
10653	18/08/1963	16/06/2016	M	1			09/05/2016	19/02/2016		475	0	0	0	0	0	0	0	0		0	0	0		0	0	0	0	0	0	0	1	0	0	0	1	1		475	26,4	0,9	39				0	
10656	10/01/1992	14/06/2016	M	1			02/05/2016	02/03/2016		331	0	0	0	0	1	1	0	0		0	0	0		0	0	0	0	0	0	1	0	1	1	0	1	1		427	19,4	0,34	39				0	
10748	15/05/1974	23/08/2016	M	1			26/05/2016	05/04/2016		198	0	0	0	0	0	0	0	0		0	0	0		0	0	0	0	0	0	0	0	0	0	0	1	1		370	29,6	0,29	19				0	
10768	28/07/1994	09/06/2016	M	1			29/04/2016	13/04/2016		345	0	0	0	0	1	1	0	0		0	0	0		0	0	0	0	0	0	1	0	0	0	1	1	1		480	16	0,25	39				0	
10772	23/11/1967	09/06/2016	M	5			17/03/2016			55	0	0	0	0	1	0	0	0		0	0	0		0	0	1	0	0	1	1	0	0	0	0	1	1		260	9,3	0,12	51				1	
10804	31/10/1983	17/05/2016	M	1			01/01/2009	01/08/2009		846	0	0	1	0	0	0	0	0		1	0	0		1	0	0	0	0	0	0	1	0	1	0	1	1		846	36,8	0,79	0,9				0	
10808	30/07/1965	09/06/2016	M	1			01/01/2006	01/01/2007			0	9	0	0	0	0	0	0		0	0	0		0	0	0	0	0	0	0	0	0	0	0	0	0									0	
10864	21/07/1995	14/06/2016	M	1			05/07/2016	13/05/2016		519	0	0	0	0	0	0	1	1		0	0	1		0	0	0	0	0	0	0	0	1	0	0	1	1		519	23,6	0,44	119548				1	
10868	02/08/1977	21/07/2016	M	1			06/07/2016	16/05/2016		710	0	0	1	0	1	0	1	0		0	0	0		0	0	0	0	1	0	0	0	0	0	0	1	1		710	44,4	2,36	68432				0	
10904	20/07/1983	14/07/2016	M	1			01/01/2015	01/01/2008		585	0	0	1	0	1	0	0	0		0	0	0		0	0	0	0	0	0	1	1	0	0	0	1	1		585	22,5	0,34	20197				1	
